# Structural Diversity, Biological Properties and Applications of Natural Products from Cyanobacteria. A Review †

**DOI:** 10.3390/md15110354

**Published:** 2017-11-10

**Authors:** Sayed Asmat Ali Shah, Najeeb Akhter, Bibi Nazia Auckloo, Ishrat Khan, Yanbin Lu, Kuiwu Wang, Bin Wu, Yue-Wei Guo

**Affiliations:** 1 Ocean College, Zhejiang University, Hangzhou 310058, China; sayedasmat89@yahoo.com (S.A.A.S.); na_memon@yahoo.com (N.A.); naz22ia@hotmail.com (B.N.A.); ishratkhan@zju.edu.cn (I.K.); 2 Department of Applied Chemistry, Zhejiang Gongshang University, Hangzhou 310012, China; luyanbin@mail.zjgsu.edu.cn (Y.L.); wkwnpc@zjgsu.edu.cn (K.W.); 3 State Key Laboratory of Drug Research, Shanghai Institute of Materia Medica, Chinese Academy of Sciences, Shanghai 201203, China

**Keywords:** cyanobacteria, secondary metabolites, biological properties and applications

## Abstract

Nowadays, various drugs on the market are becoming more and more resistant to numerous diseases, thus declining their efficacy for treatment purposes in human beings. Antibiotic resistance is one among the top listed threat around the world which eventually urged the discovery of new potent drugs followed by an increase in the number of deaths caused by cancer due to chemotherapy resistance as well. Accordingly, marine cyanobacteria, being the oldest prokaryotic microorganisms belonging to a monophyletic group, have proven themselves as being able to generate pharmaceutically important natural products. They have long been known to produce distinct and structurally complex secondary metabolites including peptides, polyketides, alkaloids, lipids, and terpenes with potent biological properties and applications. As such, this review will focus on recently published novel compounds isolated from marine cyanobacteria along with their potential bioactivities such as antibacterial, antifungal, anticancer, anti-tuberculosis, immunosuppressive and anti-inflammatory capacities. Moreover, various structural classes, as well as their technological uses will also be discussed.

## 1. Introduction

**Figure 1:**
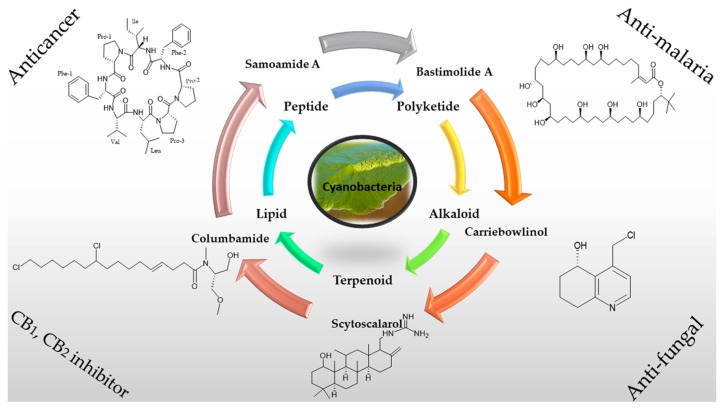
Biologically active secondary metabolites from marine cyanobacteria.

Natural products have long been a source of unique and priceless molecules with potent pharmaceutical purposes, which are waiting to be further explored [1,2,3]. In the recent era, drug discoveries from marine sources have proved themselves as significant therapeutics where the isolation of effective marine compounds classified in various chemical classes is increasing annually [4,5]. As such, one vital source of these bioactive marine secondary metabolites is from marine microbes [6,7,8,9]. Cyanobacteria, also known as blue-green algae, are considered as an important group of ancient slow-growing photosynthetic prokaryotes, which are responsible for producing atmospheric oxygen as well as play a vital role in generating a surprisingly distinct group of secondary metabolites [10]. These marine microorganisms are known to live in a different environment, which could explain the chemical diversity of the compounds that have been isolated from them. Most of these metabolites are biologically active and are produced either through the non-ribosomal polypeptide (NRP) or the hybrid polyketide-NRP biosynthetic pathways. Their structural types are important subsets of natural products possessing medical capacities such as dolastatin 10 which is known as a tubulin polymerization inhibitor, the vancomycin antibiotic, cyclosporine as an immunosuppressant, bleomycin as a chemotherapy drug, and largazole and santacruzamate A as histone deacetylase inhibitors [11,12,13]. Therefore, cyanobacteria have been regarded as a powerful source of secondary metabolites with possible technological applications in the pharmaceutical field leading to an increase in interest in these research realms [14]. In this scenario, until now an assortment of secondary metabolites such as peptides, polyketides, alkaloids, lipids, and terpenes [15] from marine cyanobacteria with potent biological activities have been isolated as shown in Figure 1.

Apart from producing toxins which can be utilized as pesticides in the agricultural field due to their allelochemical nature, cyanobacteria have the ability to generate exceptional bioactive compounds that are fascinating in terms of their antibacterial, antifungal, anti-cancerous, immunosuppressive, anti-inflammatory and anti-tuberculosis activities [16,17,18,19,20,21,22]. As such, due to increased pharmaceutical values and applications, a new prospect of using cyanobacteria in the field of medicine should be upgraded, focusing on exploring new taxonomic space which may provide an advantage regarding unique compound development. As such, a large number of secondary metabolite productions from cyanobacteria have incited investigators to engineer these organisms in order to achieve highest production. For example, the first cyanobacterium *Synechocystis* sp. PCC 6803 underwent full genome sequencing by Kaneko et al., in 1996 [23]. Recently, 83% enhancement was observed in ethanol production after the pyruvate carboxylase enzyme in *Synechocystis* sp. PCC 6803 was engineered [24]. Moreover, a project named CyanoGEBA (Genomic encyclopedia of Bacteria and Archea) was devised for genome sequencing for the identification of biosynthetic gene clusters of isolated known compounds [25] which revealed that cyanobacteria should be highlighted due its ability to encode for several metabolite gene clusters. On the other hand, the advancement in analytical HPLC, NMR spectroscopy, high throughput/high content screening and the hyphenated techniques, together play a significant role in promoting the new bioactive compounds [26].

The intensification of marine natural product study will likely become more notable, with the addition of innovative technologies which may help for the isolation of compounds produced in scarce amount as well as the capacity to identify their structures. Comprehensively, state-of-the-art technologies along with effective collaborations between academics and industrial research will be indispensable to assure the prospective success of marine cyanobacterial secondary metabolites as unique and groundbreaking medicinal entities for the therapy of human disease. In this article, an overview of various isolated secondary metabolites from cyanobacteria classified into different structural classes such as peptides, polyketides, alkaloids, and lipids along with their biological properties and applications are discussed. That is, approximately one hundred recently published compounds with their structural diversity and absolute configuration including highlighted residues are given. These reported studies have great possibilities in contributing to the understanding and development of marine cyanobacterial natural products.

## 2. Chemical Diversity of Secondary Metabolites from Cyanobacteria

The necessity of new compounds to be utilized as drugs has always been on the first row in pharmaceutical industries. Secondary metabolites that are isolated from cyanobacteria are of greater involvement due to their unique structural scaffolds and competency to produce potent drugs with significant biological properties such as antibacterial, antifungal, anticancer, anti-tuberculosis, immunosuppressive, and anti-inflammatory. According to Vijayakumar et al. and Mi et al., most of the secondary metabolites have been extracted from the genera *Oscillatoriales, Lyngbya, Mooria, Okeania* and *Caldora* [27,28]. However, the genera *Moorea* and *Okeania* were previously recognized, as has the polyphyly genus *Lyngbya*, while the genus *Caldora* was distinguished as *Symploca*. Until now, approximately 58% metabolites are reported from *Oscillatoriales* while 35% of natural products are reported from the genus *Lyngbya*. Therefore, marine cyanobacteria have attracted scientists from diverse areas including medicinal chemistry, pharmacology and marine chemical ecology. The different chemical classes, as well as potent biological properties of secondary metabolites isolated from cyanobacteria, are elaborated below.

### 2.1. Peptides

Bioactive constituents from marine cyanobacteria have boosted attention in marine natural products research. The wide biologically active range of marine peptides from peptides–polyketides biosynthetic origin has high therapeutic potential which attracted pharmaceutical industries [29]. Study of these marine cyanobacteria has resulted in the development of a wealth of biologically active secondary metabolites, and the preponderance of these are peptides and peptide-derived compounds. Distinct structural classes of peptides such as linear peptides, linear depsipeptides, linear lipopeptides, cyclic peptides, cyclic depsipeptides, and cyclic lipopeptides have been previously discovered from marine cyanobacteria [30]. Notably, some cyanobacterial peptides and related hybrid metabolites have even proceeded as therapeutic lead compounds [20,29]. For example, brentuximab vedotin (trade name Adcetris), a marine peptide-derived medicine, was approved by the U.S. Food and Drug Administration (FDA) in 2011 for the treatment of Hodgkin lymphoma and anaplastic large cell lymphoma [31]. A summary of recently published secondary metabolites isolated from marine cyanobacteria is discussed below.

#### 2.1.1. Cyclic Peptide

Urumamide (**1**), a cyclic depsipeptide, was isolated from the marine cyanobacteria *Okeania* sp. collected from Ikei Island, Okinawa [32]. With accordance to the spectral analysis, urumamide contained seven *α*-amino acids (proline, valine, leucine, *N*-Me-isoleucine, *N*-Me-alanine, *N*-Me-leucine, and *N*-Me-valine), one *α*-hydroxy acid, a 2-hydroxy-isovaleric acid (Hiva), and one *β*-amino acid, (2*S*,3*R*)-Map. The absolute configuration of *α*-hydroxy acids (Hiva) was deduced by chiral HPLC analysis, assigning the stereochemistry as d-Hiva, while all *α*-amino acid residues were assigned l-form configuration using Marfey’s method. Urumamide (**1**) exhibited weak growth-inhibitory activity against human cancer cells with IC_50_ values of 18 ± 0.5 µM and 13 ± 0.5 µM, respectively (*n* = 3) as well as inhibited chymotrypsin with an IC_50_ value of 33 ± 9 µM (*n* = 3). Cyclic depsipeptide medusamide A (**2**), was isolated from a new genus of marine cyanobacterium, collected from Coiba Island on the Pacific coast of Panama [33]. Spectral analysis revealed the presence of four *β*-amino acids, one *α*-amino acid, and two *α*-hydroxy acid residues followed by representative residues being Amha 1–4, valine (Val), leucic acid (OLeu), and *α*-hydroxyisovaleric acid (Hiva). Subsequently, the absolute configuration of hydroxy acids deduced by chiral HPLC while using Marfey’s method for valine and Amha residues confirmed the stereochemistry as l-Hiva, d-OLeu, d-Valine and (2*R*,3*R*)-Amha respectively. Medusamide A (**2**) was found to be inactive against cancer cell growth. Odoamide (**3**), a cyclic depsipeptide containing a 26-membered ring was isolated from an Okinawan cyanobacterium genus *Okeania* sp. from Japan [34]. The spectral analysis suggested the presence of *N*-methyl alanine, isoleucine, *N*-methyl glycine, *N*-methyl phenylalanine, alanine, and 2-hydroxy-3-methylpentanoic acid (Hmpa) while the remaining structure was identified as 5,7-dihydroxy-2,6,8-trimethyl-undec-2-enoic acid (Dtuea). The absolute configuration of *N*-Me-Ala, Ile, *N*-Me-Phe and Ala residues were deduced by applying Marfey’s method and HPLC analysis, showing the arrangements as l, l, d, and l, respectively, and d-allo for Hmpa. Odoamide (**3**) showed moderate toxicity against brine shrimp with an LD_50_ value of 1.2 µM and potent cytotoxicity activity against HeLa S3 cells with an IC_50_ value of 26.3 nM.

**Figure 2:**
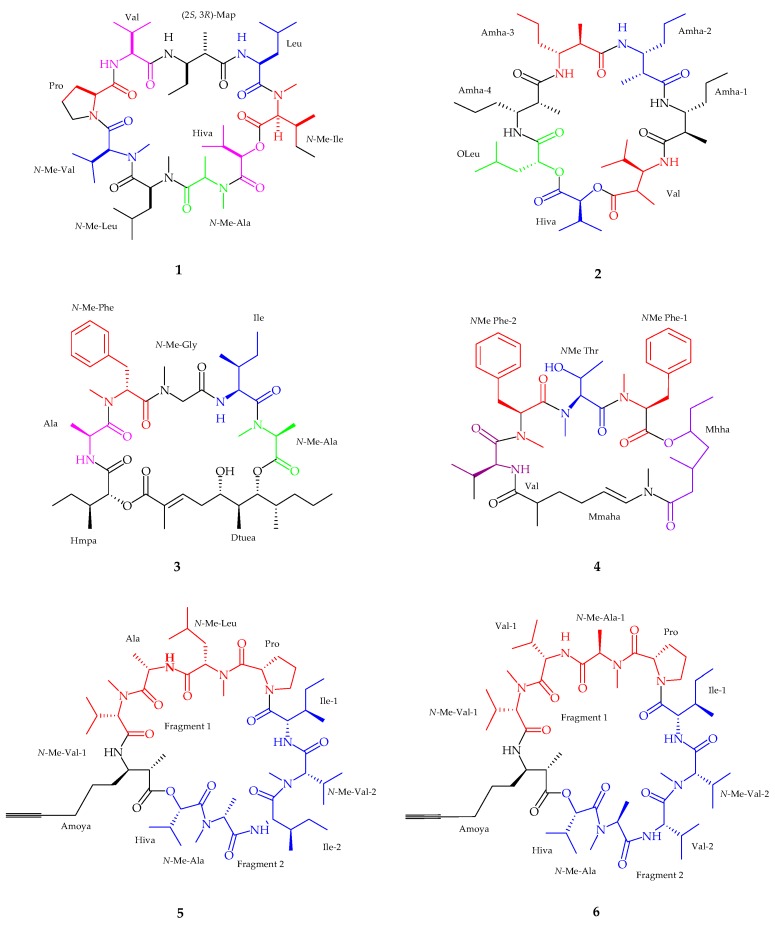
The chemical structures of: uramamide (**1**); medusamide A (**2**); odoamide (**3**); bouillonamide (**4**); companeramide A (**5**); and companeramide B (**6**).

A cyclic depsipeptide, bouillonamide (**4**), was extracted from *Moorea bouillonii* collected from the northern coast of New Britain, Papua New Guinea [35]. Six partial structures were generated using spectroscopic analysis, which included two *N*-methyl phenylalanine residues, one *N*-methyl threonine residue, one valine residue, a 2-methyl-6-methylamino-hex-5-enoic acid (Mmaha) moiety, and a 3-methyl-5-hydroxy heptanoic acid (Mhha) unit. *N*-MePhe and Val residues were detected by Marfey’s method showing l-forms amino acids. Bouillonamide (**4**) showed moderate cytotoxic activity (IC_50_ of 6.0 μM) against neuro-2a mouse neuroblastoma cell line. Two new cyclic depsipeptides, companeramides A (**5**) and B (**6**), were isolated from marine cyanobacterial assemblage in Coiba National Park, Panama [36]. The presence of two *N*-methyl valine, one *N*-methyl leucine, one *N*-methyl alanine, one alanine (Ala), one proline (Pro) and two isoleucine (Ile) amino acid residues along with one hydroxyisovaleric acid (Hiva) followed by the presence of 3-amino-2-methyl-7-octynoic acid (Amoya) were confirmed by spectral analysis. On the other hand, companeramide B (**6**) revealed a similar structural composition to companeramide A (**5**), comprising of existing residues like Amoya, two *N*-Me-Val, Pro, Ile, and Hiva with a major difference suggesting that (**6**) had two Val spin systems. The absolute configuration of companeramide A (**5**) and B (**6**) residues were deduced as l-configuration for Ala, *N*-Me-Ala, Pro and both Ile, *N*-Me-Leu, *N*-Me-Val, and *S*-Hiva for companeramide A (**5**) while an l- and d-configuration was established as *N*-Me-l-Val, l-Val, *N*-Me-d-Ala (fragment 1), *N*-Me-l-Ala (fragment 2), and l-Pro for companeramide B (**6**). Companeramides A (**5**) and B (**6**) showed moderate anti-plasmodial activity (Figure 2, Table 1).

Dudawalamides A–D (**7**–**10**), Dhoya-containing cyclic depsipeptides belonging to the kulolide superfamily, were isolated from *Moorea producens*, collected from Dudawali Bay, Papua New Guinea [37]. Dudawalamides A (**7**) consisted of six partial structures of amino acids and one Dhoya unit such as glycine, *N*-methyl-phenylalanine, *N*-Me-isoleucine, proline, alanine, lactic acid and a 2,2-dimethyl-3-hydroxy-7-octynoic acid (Dhoya). Moreover, the sequence of the residues were identified as cyclo-[Pro-*N*-Me-Phe-Gly-Dhoya-Lac-Ala-*N*-Me-Ile]. The absolute configuration was revealed as l-configuration for all residues like Lac, Ala, *N*-Me-Ile, and Pro, while *R* was assigned for Dhoya with an unusual d-configuration for the remaining residue *N*-Me-Phe. Dudawalamide B (**8**), was similar to dudawalamide A (**7**) with the only difference lies in the replacement of glycine, isoleucine, and lactic acid units in dudawalamide A by valine, and *N*,*O*-dimethyltyrosin units. The sequence of the residues were identified as cyclo-[Dhoya-Val-*N*-Me-Phe-Pro-*N*,*O*-diMe-Tyr-Ala]. Dudawalamide C (**9**) and D (**10**) were also an analog of dudawalamide A and were closely similar to each other. Both structures of dudawalamide C and D had similar amino acid residues excluding a hydroxy unit, such as Dhoya, valine, proline, *N*-methylphenylalanine, and glycine with a prominent difference showing the presence of a hydroxyisovaleric acid (Hiva) residue in dudawalamide C (**9**) and a 2-hydroxy-3-methylpentanoic acid (Hmpa) unit in dudawalamide D (**10**). The sequence of the residues was established as cyclo-[Dhoya-Val-*N*-Me-Val-Hiva-Pro-*N*-Me-Phe-Gly] and cyclo-[Dhoya-Val-*N*-Me-Val-Hmpa-Pro-*N*-Me-Phe-Gly], respectively. The unit Dhoya was deduced as *S-*configuration while the other amino acid residues as l-configuration and a hydroxy unit Hiva was established as l while the Hmpa unit was determined to be d-allo. Dudawalamide A (**7**) and D (**10**) showed potent activities against *Plasmodium falciparum* with IC_50_ values of 3.6 and 3.5 μM respectively. Moreover, D (**8**) showed effective activity against *Leishmania donovani* with an IC_50_ value of 2.6 μM. New cyclic depsipeptides, namely kohamamides A–C (**11**–**13**), belonging to the superfamily kulolide, isolated from a cyanobacterium *Okeania* sp. and was collected in Japan [38]. Kohamamide A (**11**) was seen to contain five amino acids residues with two hydroxy units including alanine (Ala), leucine (Leu), proline (Pro), valine (Val), methyl ester valine (*N*-Me-Val), dimethyl hydroxy octynoic acid (Dhoya) and 2-hydroxy-3-methylpentanoic acid (Hmpa) with a sequence identified as cyclo-[Dhoya-Ala-Leu-Pro-Hmpa-*N*-Me-Val-Val]. The spectral analysis also revealed that the Kohamamides B and C (**12** and **13**) were closely similar to A (**11**) excluding the difference for the degree of unsaturation of the Dhoya moiety. Kohamamide B (**12**) showed potent activity against human cancer cells HL60 with an IC_50_ value of 6.0 ± 2.1. The kohamamides A (**11**) and C (**13**) showed weak activity against HL60 cells with IC_50_ values of 19 ± 3 and 11 ± 5, respectively.

**Figure 3:**
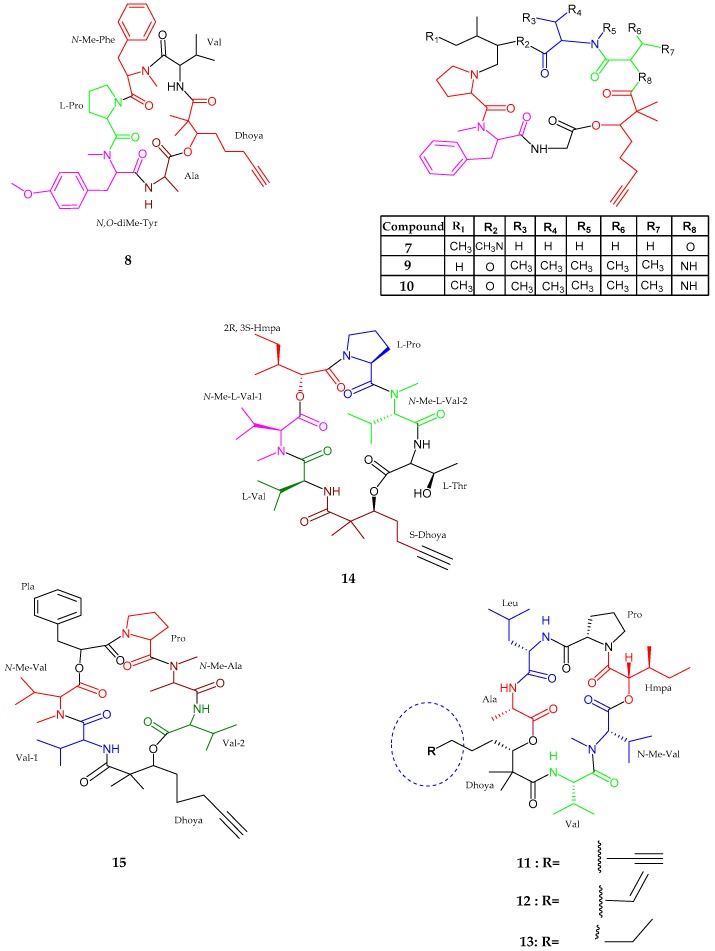
The chemical structures of: dudawalamides A–D (**7**–**10**); kohamamides A–C (**11**–**13**); and viequeamides A (**14**) and B (**15**).

Viequeamides A (**14**) and B (**15**), a Dhoya-containing cyclic depsipeptides, were isolated from *Rivularia* sp. near Puerto Rico [39]. Viequeamide A (**14**) consisted of five partial structures of amino residues such as threonine, proline, valine, and two methyl esters valine along with Dhoya and Hmpa moieties, with the sequence cyclo-[Dhoya–Val-*N*-Me-Val-1–Hmpa–Pro-*N*-Me-Val-2-Thr]. The stereochemistry was established for all amino acid units as l-configuration, while Hmpa unit was established to be 2*S*, 3*R*, along with Dhoya unit bearing *S* configuration. The difference observed in B (**15**) was the presence of aryl protons, phenyl lactic acid (Pla), and *N*-methyl alanine. The stereochemistry for amino acid units was proven to be l-configuration, while *S* configuration was assigned for phenyl lactic acid and Dhoya units. Viequeamide A (**14**) showed potent cytotoxic activity against H460 human lung cancer cells with an IC_50_ value of 60 ± 10 nM (Figure 3, Table 1).

A cyclic depsipeptide named pitiprolamide (**16**), which was noted to be structurally related to dolastatin 16, along with four pitipeptolides C–F (**17**–**20**), which are analogs of pitipeptolides A and B, were isolated from *Lyngbya majuscula* collected from Piti Bomb Holes, Guam [40,41]. Pitiprolamide (**16**) comprised eight amino acid residues, namely proline 1–4 (Pro), valine (Val), dolaphenvaline (Dpv), 2-hydroxy isovaleric acid (Hiva) and a unit of 2,2-dimethyl-3-hydroxyhexanoic acid (Dmhha), bearing the sequence as Pro1-Dpv-Pro2-Pro3-Hiva-Pro4-Val-Dmhha. The absolute configuration was revealed as l-configuration for all proline units and Val unit, while Dmhha unit showed 3*R*, Hiva unit had an *S* and Dpv unit bear 2*S*, 3*R* configuration. Pitipeptolide C (**17**) was a tetrahydro-analog of previously published pitipeptolide A [41] which consisted of five amino acid residues such as valine, *N-*methyl phenylalanine, proline, isoleucine, and glycine including one *α*-hydroxyl methylpentanoic acid (Hmpa) with the completely saturated fatty acid derived unit 2,2-dimethyl-3-hydroxy oxtanoic acid (Dhoaa). Pitipeptolide D (**18**) was an analog of lower degree methylation of pitipeptolide A, containing the same residues as pitipeptolide C (**17**) excluding *N*-methyl group modification. Pitipeptolide E (**19**) and F (**20**) showed close similarity to each other with one missing methylene group. Moreover, pitipeptolide E (**19**) had Hmpa → Hiva displacement, while pitipeptolide F (**20**) had Ile → Val movement compared to pitipeptolide. All amino and *α*-hydroxy acids showed l-configuration while it was proved that the fatty acid derived units showed *S* configuration as compared to pitipeptolide A. Pitiprolamide (**16**) showed weak cytotoxic activity against HCT116 colorectal carcinoma (IC_50_ 33 μM) and MCF7 breast adenocarcinoma cell lines (IC_50_ 33 μM). Moreover, pitiprolamide F (**20**) showed the highest potency in the disc diffusion assay against *Mycobacterium tuberculosis*.

**Figure 4:**
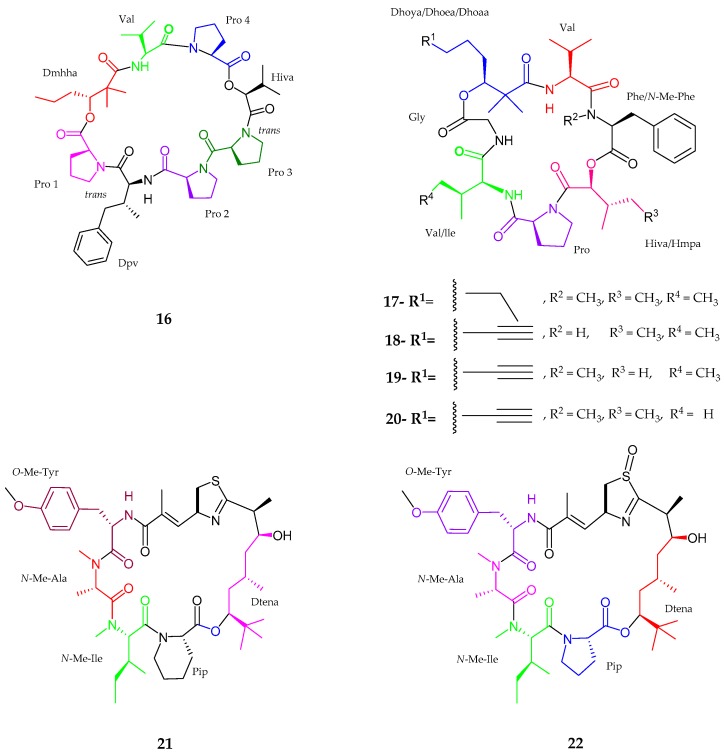
The chemical structures of: pitiprolamide (**16**); pitipeptolides C–F (**17**–**20**); apratoxin H (**21**); and apratoxin A sulfoxide (**22**).

Thornburg et al. [42] discovered new apratoxin analogs, namely, apratoxin H (**21**) and apratoxin A sulfoxide (**22**), from *Moorea producens,* in the Gulf of Aqaba, Red Sea. Both apratoxin H (**21**) and apratoxin A sulfoxide (**22**) were closely similar to the previously isolated compound apratoxin A with a difference in the replacement of proline residue in apratoxin A by pipecolic acid (Pip) in apratoxin H (**21**). As for apratoxin A sulfoxide (**22**), spectral analysis confirmed the presence of oxidized sulfur atom within the thiazoline residue. Marfay’s analysis showed the presence of *N*-Me-l-Ala, d-Cya for an *S* configuration at *α* carbon, *N*-Me-l-Ile, and *O*-Me-l-Tyr for both compounds. Additionally, l-Pip was seen for apratoxin H unit and l-Pro for apratoxin A sulfoxide. Apratoxin H (**21**) showed significant cytotoxicity to human NCI-H460 lung cancer cells with an IC_50_ value of 3.4 nM, while an IC_50_ value of 89.9 nM was noted for apratoxin A sulfoxide (**22**), revealing a nearly 36-fold reduction in cytotoxicity. Therefore, these results showed that apratoxin cytotoxicity was sensitive to certain modifications of the moCys thiazoline unit (Figure 4, Table 1).

**Figure 5:**
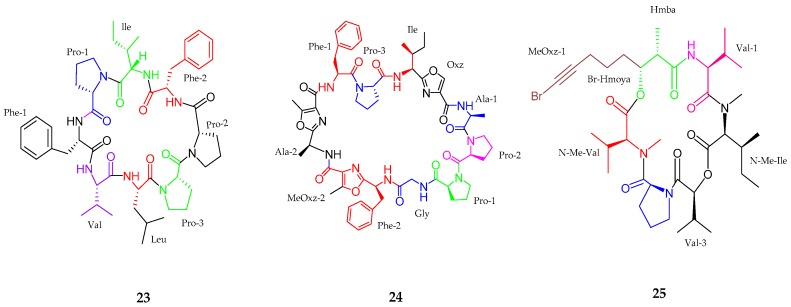
The chemical structures of: samoamide A (**23**), wewakzole B (**24**) and odobromoamide (**25**).

A new bioactive cyclo octapeptide, samoamide A (**23**) was purified from *Symploca* sp. collected in American Samoa in 2017 [43]. The planar structure of samoamide A (**23**) consisted of eight proteogenic and unmodified amino acid residues and finally was resolved as cyclic Leu-Val-Phe(1)-Pro(1)-Ile-Phe(2)-Pro(2)-Pro(3) (cyc-LVFPIFPP). All amino residues were revealed to be in l-configuration. The proline residues were assigned as *trans* (Pro-1), *cis* (Pro-2) and *trans* (Pro-3) due to their difference in chemical shifts between the *β-* and *γ-*carbons. Samoamide A (**23**) was tested against a series of cancer cell line in vitro, including NCI H-460 human non-small-cell lung cancer cells with an IC_50_ value of 1.1 μM, HCT-116 human colorectal carcinoma cells with an IC_50_ value of 4.5 μM, H125 human lung adenocarcinoma, MCF7 human breast adenocarcinoma, LNCaP human prostate cancer, OVC5 human ovarian cancer, U251N human glioblastoma, PANC-1 human pancreatic carcinoma epithelial like cells, CEM human acute lymphoblastic leukemia, and CFU-GM progenitor cells of human granulocytic and monocytic lineages. Samoamide A (**23**) was found broadly cytotoxic to many cancer lines without any remarkable selectivity. Lopez et al. [44] reported a cyclic peptide, wewakzole B (**24**), isolated from *Moorea producens* near Jeddah, Saudi Arabia. Wewakzole B was closely similar to wewakzole and most likely belonged to the cyanobactin class [45]. Wewakzole B (**24**) consisted of nine basic and three modified amino acid residues, including glycine (Gly), isoleucine (Ile), alanine (Ala × 2), phenylalanine (Phe × 2), proline (Pro × 3), oxazole (Oxz), and methyloxazole (MeOxz × 2). The absolute configuration analysis revealed that alanine, phenylalanine, proline, and isoleucine residues were in the l-configuration. Wewakzole B (**24**) showed potent toxicity against human H460 lung cancer cells (IC_50_ 1.0 μM) and moderate toxicity against human MCF7 breast cancer cells (IC_50_ 0.58 μM). Odobromoamide (**25**), a terminal alkynyl bromide-containing cyclodepsipeptide was isolated from *Okeania* sp. in Japan [46]. The sequence of odobromoamide (**25**) was arranged as *N*-Me-Val-Pro-Val-*N*-Me-Ile-Hmba-Br-(Hmoya) where the absolute configuration was seen to be l-form for all amino acid and Hmba residues. Finally, the stereostructure of odobromoamide (**25**) was deduced as 2*S*, 8*S*, 13*S*, 18*S*, 19*S*, 25*S*, 30*S* and 31*R*. Odobromoamide (**25**) exhibited potent cytotoxic activity against HeLa S3 cells with an IC_50_ value of 0.31 μM (Figure 5, Table 1).

**Figure 6:**
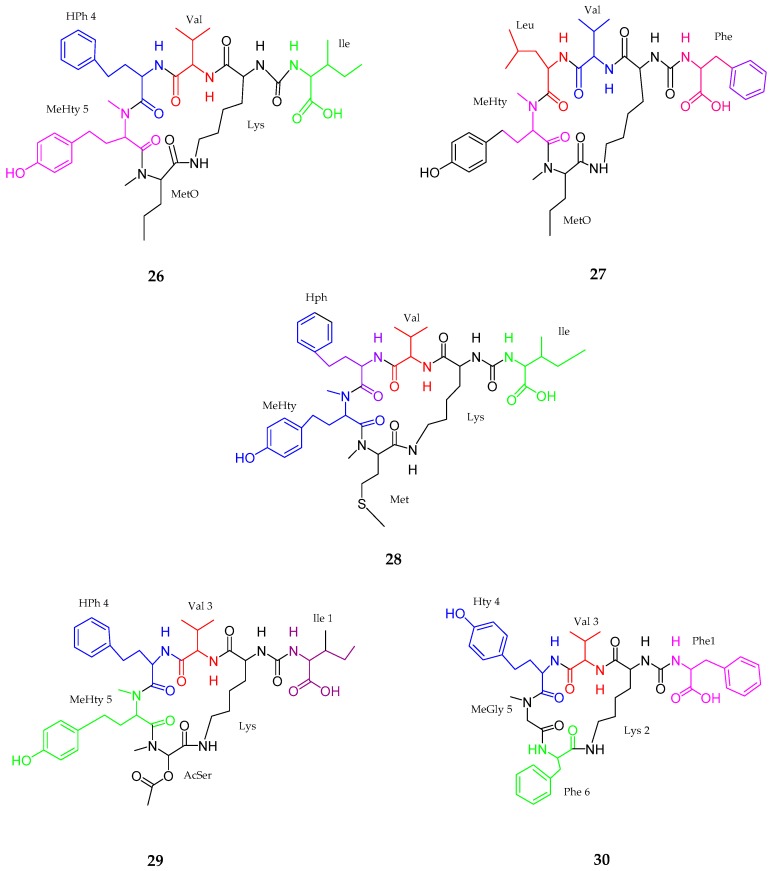
The chemical structures of anabaenopeptins (**26**–**30**).

Five new cyclic hexapeptides, anabaenopeptins (**26**–**30**), was isolated from the extract of cyanobacterial bloom material composed of Nodularia spumigena, Aphanizomenon flos-aquae and *Dolichospermum* sp. from the Baltic Sea [47]. According to mass spectrum analysis, anabaenopeptin NP 883 (**26**) contained (Ile + CO [Lys + Val+ Hph + MeHty + MetO]), anabaenopeptin NP 869 (**27**) contained (Phe + CO [Lys + Val+ Leu + MeHty + MetO]), anabaenopeptin NP 867 (**28**) contained (Ile + CO[Lys + Val + Hph + MeHty + Met]), anabaenopeptin NP 865 (**29**) contained (Ile + CO[Lys + Val + Hph + MeHty + AcSer]) and anabaenopeptin NP 813 (**30**) contained (Phe + CO[Lys + Val + Hty + MeGly + Phe]). Most of them belonged to the subclass nodulapeptins when considering Met, Meto or AcSer in position 6. No activity was determined for these compounds (Figure 6, Table 1).

#### 2.1.2. Linear Peptides

A linear depsipeptide, maedamide (**31**), isolated from the species of *Lyngbya,* was collected from Okinawa Prefecture [48]. Maedamide retained many important amino acid residues such as a 4-amino-3-hydroxy-5-phenylpentanoic acid (Ahppa), allo-d-isoleucine, and *N*-Me-d-phenylalanine, along with two hydroxy acid moieties. Spectral analysis revealed the presence of valic acid, isoleucic acid, proline, isoleucine, glycine, *N*-Me-phenylalanine, and *O*-Me-proline while chiral HPLC established the stereochemistry as l, allo-d, l, allo-d, d, and l, respectively. Maedamide (**31**) inhibited the growth of human cancer cells and selectively inhibited chymotrypsin with the IC_50_ value of 45 µM. Meanwhile, maedamide showed strong protease inhibitory activity against chymotrypsin. A linear pentapeptide, caldoramide (**32**), an analog of belamide A and dolastatin 15 (**33**), was isolated from *Caldora penicillata* in Florida [49]. Caldoramide consisted of one *O*Me, *N,N*-dimethylvaline, valine, *N*-Me valine, *N*-Me isoleucine, a putative phenylalanine and a conjugated enol ether residues. The combined data established the structure of caldoramide as *N,N*-diMe-Val-Val-*N*Me-Ile-3-*O*-Me-4-benzylpyrrolinone and the l-configuration observed for all amino acids. Caldoramide (**32**) displayed anti-proliferative activity upon cells including oncogenic KRAS or HIF transcription factors, namely, parental HCT 116 over HCT 116^HIF^^-1*α*−/−HIF^^-2*α*−/−^ and HCT 116^WT KRAS^. Three lipopeptides, tasiamides C–E (**33**–**35**), were isolated from *Symploca* sp. near Kimbe Bay, Papua New Guinea [50]. Tasiamide C (**33**) consisted of five amino and two hydroxy acid residues like proline methyl ester (*N*-MePro), phenylalanine methyl ester (*N*-MePhe), alanine (Ala), isoleucine (Ile), glutamine methyl ester (*N*-MeGln), and two 2-hydroxy-3-methylbutyric acids (Hmba), indicating an overall linear arrangement. Three amino acids (Pro, Ala and *N*-MeGln) were L-configuration, *N*-MePhe was d-configuration and d-allo configuration of Ile residue was assigned for tasiamide C. The two Hmba stereo-centers suggested that the absolute configuration of this compound was 2*S*, 8*R*, 18*S*, 21*R*, 22*S*, 27*S*, 33*R*, and 38*S*. Tesiamide D (**34**) resulted in the loss of *N*-methyl on the phenylalanine (Phe) residue, thus proving an arrangement of *N*-MePro, Phe, Ala, Ile, *N*-MeGln, Hmba-1, and Hmba-2 with the absolute configuration being 2*S*, 8*R*, 17*S*, 20*S*, 21*S*, 26*S*, 32*R*, and 37*S*. The presence of one additional amino acid residue leucine (Leu) and a hydroxyl unit 2-hydroxy-4-methylpentanoic acid (H4mpa) in tesiamide E (**35**) differentiated it from tesiamide C and D with the absolute configuration being 2*S*, 8*R*, 20*S*, 21*S*, 26*S*, 32*S*, and 38*S*.

**Figure 7:**
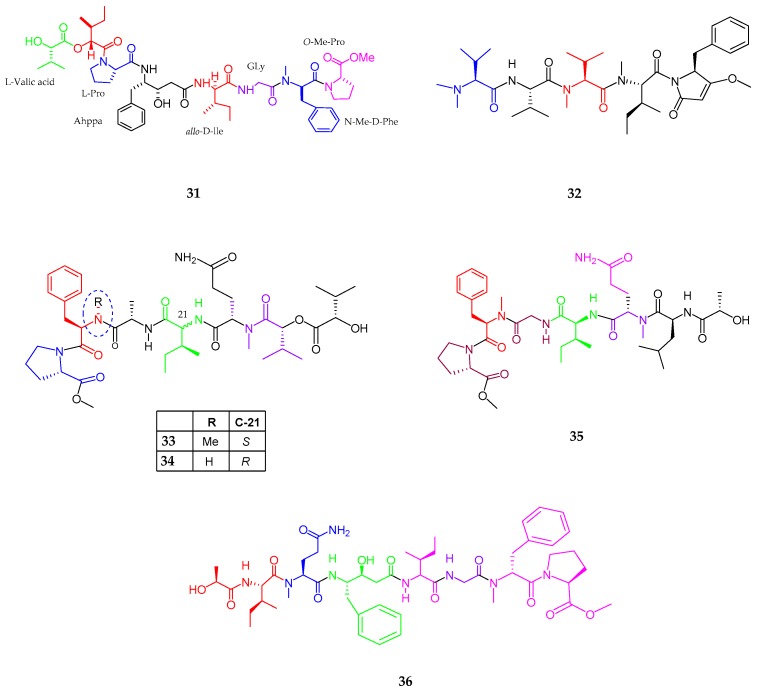
The chemical structures of: maedamide (**31**); caldoramide (**32**); tasiamides C–E (**33**–**35**); and tasiamide F (**36**).

Tesiamide F (**36**), an analog of tesiamide B, was isolated from cyanobacterial species *Lyngbya*, from Guam [51]. As compound tesiamide F was an analog of the previously isolated tesiamide B, the difference lies in the replacement of amino acid residues in tesiamide B to Ala → Gly, Leu → Ile, Val → Ile, with the presence of a statin unit Ahppa (Amino hydroxyphenyl pentanoic acid) differing it from other tesiamide structures. Tasiamides C (**33**) and D (**34**) were found to be inactive against the HCT-116 colon cancer cell line (tasiamide C, IC_50_ > 25 μM; tasiamide D, IC_50_ ≈ 25 μM). The anti-proteolytic activity of tesiamide F (**36**) was evaluated in vitro against cathepsins D, E, and BACE1 (an enzyme involved in the pathogenesis of Alzheimer’s disease), which revealed a low nanomolar inhibitory activity. Compound tesiamide F was considered less potent against BACE1. Additionally, tasiamide and tasiamide B showed moderate cytotoxicity against KB cells, with IC_50_ values of 0.48 and 0.8 μM, respectively (Figure 7, Table 1).

**Figure 8:**
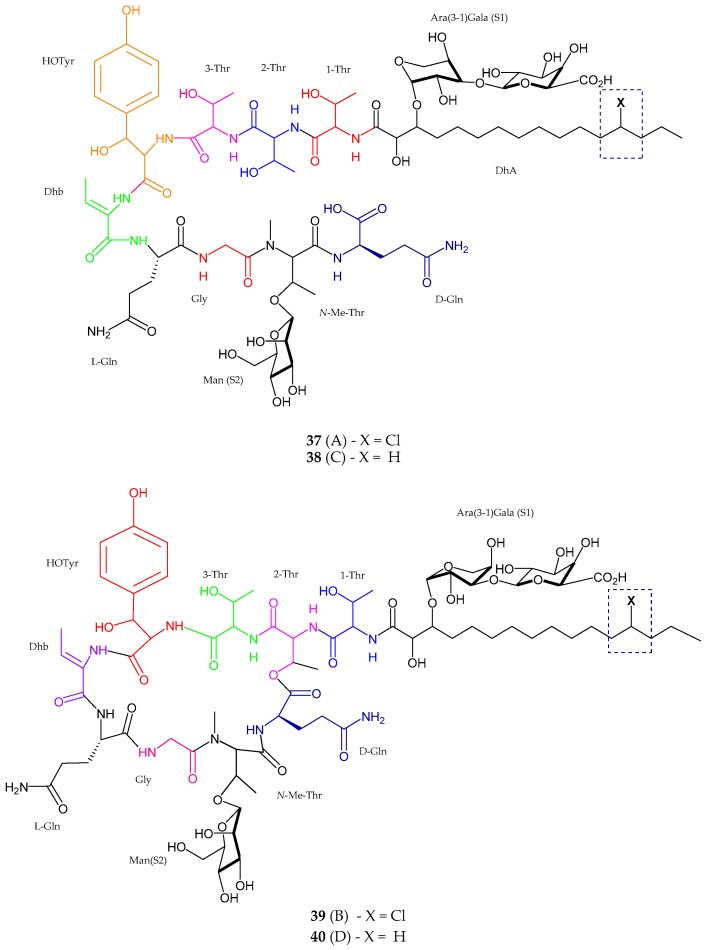
The chemical structure of balticidins A–D (**37**–**40**).

Two new antifungal linear lipopeptides, balticidin A (**37**) and C (**38**), and two new cyclic analog balticidin B (**39**) and D (**40**) were extracted from *Anabaena cylindrica* collected from the Baltic Sea, Rügen Island, Germany [52]. Balticidin A consisted of nine amino acid residues such as threonine (Thr) (Thr × 3), dehydroamino butyric acid (Dhb), glutamic acid (Glx) (Glx × 2), glycine (Gly), *β*-hydroxytyrosine (possessed AA’BB’ spin system), *N*-Me-Thr (spin system lacking an amide proton) and *β*-HOTyr. Spectrometric data also revealed the presence of chlorinated dihydroxy (10-DhA) fatty acid moiety and three anomeric protons which proved the presence of three sugar moieties. The configuration of these monosaccharides was established as d(+)-mannose, d(−)-arabinose and d(+)-galacturonic acid. Balticidin C (**38**) showed the difference in an aliphatic chain where it contained hydrogen instead of chlorine in the side chain attached to the first threonine unit. Balticidin D (**40**) was seen to be in close similarity with balticidin B showing the difference in hydrogen excluding the chlorine on the fatty acid residue. Balticidins A–D (**37**–**40**) showed active inhibition zone against *Candida maltose* with 12, 15, 9 and 18 mm, respectively (Figure 8, Table 1).

**Figure 9:**
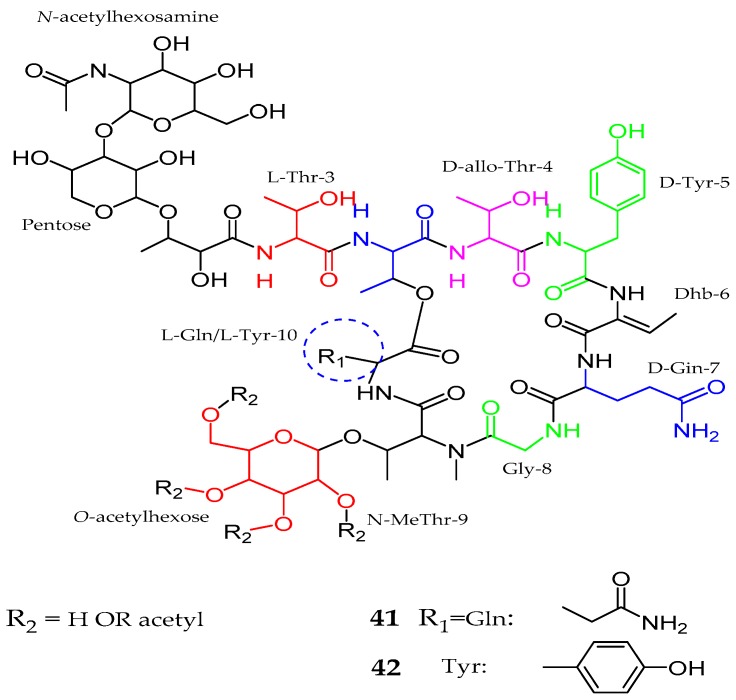
Chemical structure of Hassallidins C–D (**41**–**42**).

In 2014, a draft genome of *Anabaena* sp. SYKE748A was obtained and reported as responsible for the production of numerous unique glycosylated lipopeptides [53]. Thus, hassallidins C (**41**) and D (**42**), which resemble previously isolated antifungal hassallidins A and B, separated from an epilithic cyanobacterium *Hassallia* sp. [54]. Spectral analysis confirmed the presence of nine amino acid residues, namely, threonine (Thr × 3), tyrosine (Tyr × 2), glutamine (Gln × 2), glycine (Gly), and *N*-methyl threonine (*N*-MeThr) along with one 2,3-dihydroxy fatty acid, and three sugar moieties with configuration l-Thr, d-Thr, d-allo-Thr, d-Tyr, d-Gln, Gly, *N*-MeThr, l-Gln, and l-Tyr. The main difference in both structures was observed at position 10, as in hassallidin C (**41**) with glutamine (l-Gln) while hassallidin D (**42**) with tyrosine (l-Tyr). Both compounds inhibited the growth of *Candida albicans*. Hassallidin D was active against *Candida albicans* and *Candida krusei* with MIC value ≤ 2.8 μg/mL (1.5 μM). Interestingly, the linear form of hassallidin C had an MIC of ≤36 μg/mL (20 μM), indicating the significance of the ring structure for antifungal activities (Figure 9, Table 1).

A new acetylene-containing lipopeptide, kurahyne (**43**), was obtained from a cyanobacterial collection that mostly consisted of *Lyngbya* sp. from Kuraha, Okinawa [55]. Kurahyne (**43**) contained seven residues including one proline, two *N*-methyl valines, two *N*-methyl isoleucines, one 2-(1-oxo-propyl)-pyrrolidine (Opp) and a 2-methyloct-2-en-7-ynoic acid (Moya). The stereochemistry indicated l-configuration for all amino acids in kurahyne, while the absolute configuration in Opp moiety was elucidated to be 4*S*. Kurahyne was observed to hinder the growth of both HeLa cells and HL60 cells with IC_50_ values of 3.9 ± 1.1 μM and 1.5 ± 0.1 μM sequentially. Kurahyne B (**44**), a new analog of kurahyne (**43**), was isolated from the marine cyanobacterium *Okeania* sp. near Jahana, Okinawa [56]. Kurahyne B was seen to have one *N*-methylisoleucine and one isoleucine, while kurahyne (**43**) had two *N*-methylisoleucines. Kurahyne B (**44**) repressed the growth of human cancer cells to the same extent as kurahyne (**43**). An acetylene containing lipopeptide, Jahanyne (**45**) was isolated from *Lyngbya* sp. near Jahana, Okinawa [57]. The structure consisted of seven amino acid residues with one 2-(1-oxo-ethyl)-pyrrolidine unit (Oep) and 2,4-dimethyldec-9-ynoic acid (fatty acid unit). The sequence of jahanyne was arranged as Pro1-Oep-*N*-Me-Phe-*N*-Me-Val1-*N*-Me-Val2-*N*-Me-Val3-Pro2-*N*-Me-Ala-Fatty acid. The absolute configuration was assigned to be l form to all amino acid residues, while the Oep configuration as 3*R*. The absolute configuration of a fatty acid moiety was determined as 2*R*. Jahanyne significantly inhibited the growth of HeLa cells and HL60 cells, with IC_50_ values of 1.8 μM and 0.63 μM, respectively.

**Figure 10:**
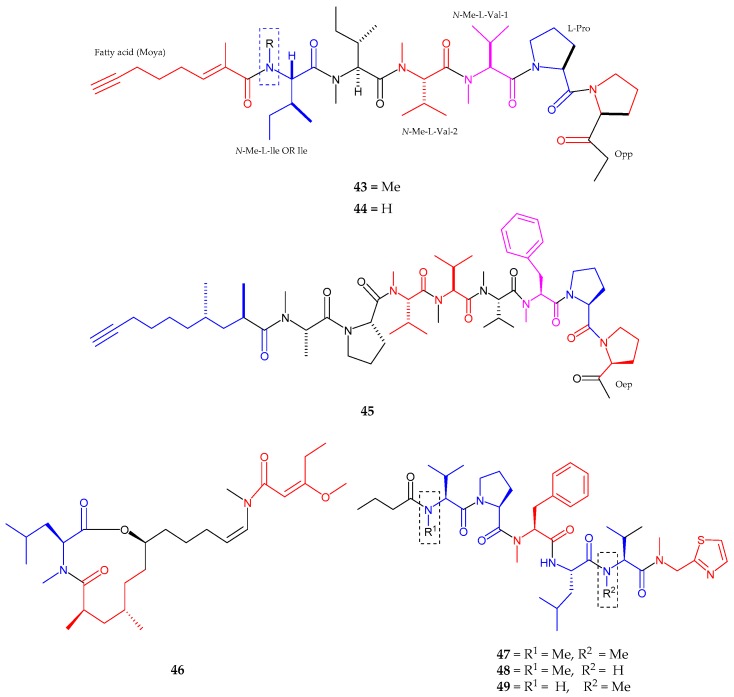
Chemical structures of: kurahyne (**43**); kurahyne B (**44**); jahanyne (**45**); and biseokeaniamides A–C (**47**–**49**).

Kanamienamide (**46**), an enamide with an enol ether 11-membered lipopeptide, isolated from *Moorea bouillonii*, was collected in Kagoshima [58]. Spectral analysis exposed the presence of eight methyl groups as well as proved the presence of three partial structures. Likewise, kanamienamide was composed of *N*-methylleucine, 7-hydroxy-2,4-dimethyl-12-(methylamino)-dodec-11-enoic acid and 3-methoxy-2-pentenoic acid. The geometries of two olefins were determined to be *Z* and *E*, respectively with the relative stereochemistry being 2’*S*,2*R*,4*S*,7*S.* Kanamienamide (**46**) exhibited growth-inhibitory activity in HeLa cells with IC_50_ 2.5 μM and caused apoptosis-like cell death in HeLa cells. It was suggested that kanamienamide (**46**) might have at least two target molecules for causing apoptosis-like cell death by upstream of caspases, downstream of caspases and other pathways. Thiazole containing lipopeptides, biseokeaniamides A–C (**47**–**49**), were identified and purified from the Japanese cyanobacterial *Okeania* sp. [59]. Biseokeaniamide A consisted of five amino acid residues including two methyl valines ester, methyl phenylalanine ester, proline, and leucine with a thiazole ring *N*-methyl-2-thiazolemethane-amine (Thz-*N*-Me-Gly) and butanoic acid (Ba). The absolute configuration of A was seen as l form for all amino acid residues. Biseokeaniamides B (**48**) and C (**49**) were found to be in close similarity to biseokeaniamide A, and were determined to be an *N*-demethyl analog of A. The difference observed in both compounds was the absence of two *N*-methyl groups. The sequence of B (**48**) was noted as Ba-*N*-Me-Val-Pro-*N*-Me-Phe-Leu-Val-Thz-*N*-Me-Gly while the sequence of C (**49**) was arranged as Ba-Val-Pro-*N*-Me-Phe-Leu-*N*-Me-Val-Thz-*N-*Me-Gly. Biseokeaniamide B exhibited growth inhibitory activity against HeLa cells with an IC_50_ value of 4.5 μM. Biseokeaniamides A–C inhibited sterol *O*-acyltransferase SOAT1 and SOAT2 with IC_50_ values of 1.8 μM, 1.3 μM, 6.9, 1.3 μM, and >12, 9.6 μM, respectively, on cellular level and with IC_50_ values of 1.8 μM, 9.6 μM, 6.8 μM, 9.9 μM, and 11, >32 μM, respectively, on enzymatic level (Figure 10, Table 1).

Furthermore, a new lipopeptide, namely maylngamide (**50**), isolated from *Moorea producens*, was collected in Hawaii [60]. Maylngamide (**50**) showed the existence of a linear alkyl group, olefin moiety along with hydroxyl group, two methoxy groups, an *N*-methyl moiety, presence of chloromethylene moiety with *Z* configuration and two *tert*-amide isomers. Malyngamide was observed active in crustacean lethality test with a LD**_100_** value of 33.30 mg/kg. Lipopeptide malyngamide Y (**51**) and a cyclic depsipeptide (+)-floridamide (**52**) were generated from *Moorea producens* from Florida [61]. A ketone group, amide group, ten methylene groups, two aliphatic methine groups, vinyl chloride atom, two methyl groups, and the methoxy group were observed by spectroscopic analysis. Malyngamide Y (**51**) exhibited cytotoxic activity against human lung cancer cell line (NCI-H460) and mouse neuro-2a neuroblastoma with an EC**_50_** value of 1.45 × 10^−5^ µM/mL. Malyngamide 4 (**53**), a new lipopeptide was isolated from *Moorea producens*, collected from the Red Sea [62]. This compound consisted of four partial structures/fragments including 7-methoxytetradec-4(*E*)-enoic acid (lyngbic acid), chloromethylene moiety along with two methylenes, olefinic methine with amide carbon and methoxy group, and *N*-substituted pyrrole-2-one substructure. Malyngamide 4 (**53**) exhibited activity against lung carcinoma (A549), colorectal carcinoma (HT-29), and breast adenocarcinoma (MDA-MB-231) with GI**_50_** values of 40, 50, and 44 µM, respectively.

**Figure 11:**
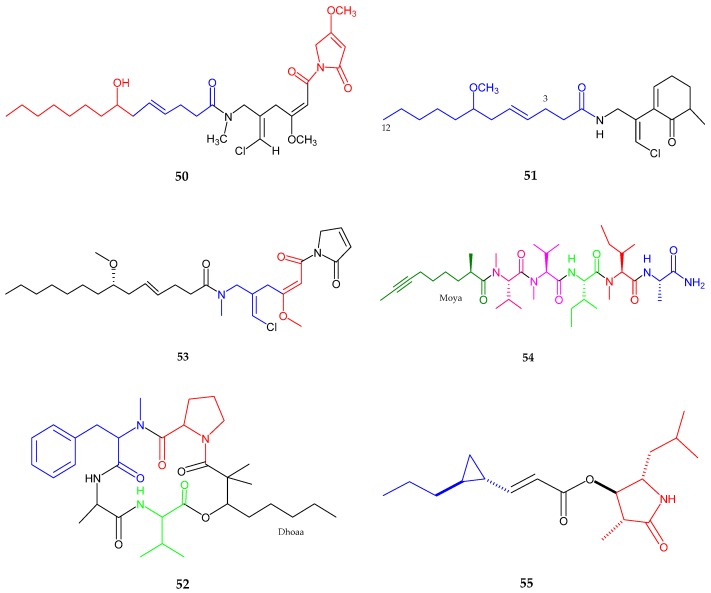
Chemical structures of: maylngamide (**50**); malyngamide Y (**51**); (+)-floridamide (**52**); malyngamide 4 (**53**); almiramide D (**54**); and hoshinolactam (**55**).

A linear lipopeptide, almiramide D (**54**), was isolated from the extract of *Oscillatoria nigroviridis*, collected from the Caribbean Sea [63]. Five amino acid residues including alanine, isoleucine, methyl isoleucine ester and two methyl valine esters together with an additional acyl moiety identified as 2-methyl-oct-7-ynoic acid (Moya) were detected by spectroscopic analysis with l form as absolute configuration. Almiramide D exhibited potent toxicity against (*Artemia salina*) nauplii with an LC**_50_** value of 3.5 μg/mL. In 2017, a new anti-trypanosomal lactam, hoshinolactam (**55**) was isolated from a marine cyanobacterium sp. collected in Okinawa [64]. Hoshinolactam possessed four methyl groups, four protons of cyclopropane ring, two olefinic protons and two carbonyl groups. Additionally, the analysis suggested the presence of two fragments derived from 4-hydroxy-5-isobutyl-3-methylpyrrolidin-2-one (HIMP) and 3-(2-propylcyclopropyl) acrylic acid (PCPA). The absolute configuration of HIMP unit was determined to be 2*R*, 3*R*, 4*S*, while 4*S*, 5*S* for PCPA unit. Hoshinolactam exhibited potent anti-trypanosomal activity against *Trypanosoma brucei* with an IC**_50_** value of 3.9 nM (Figure 11, Table 1).

### 2.2. Polyketides

**Figure 12:**
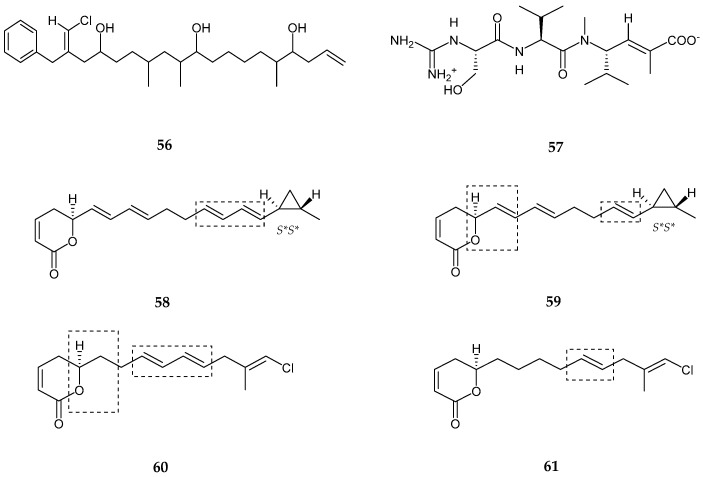
Chemical structures of: trichophycin A (**56**); cryptomaldamide (**57**); and coibacins A–D (**58**–**61**).

Trichophycin A (**56**), a vinyl chloride-containing linear polyketide, was generated from the crude extract of *Trichodesmium thiebautii* [65]. The latter consisted of three methyl groups, eleven methylenes, thirteen methines, and two quaternary carbon atoms with the presence of one aromatic ring. Trycophycin A showed several structural similarities to trichotoxin A and B [66] while possessing a longer polyketide chain than trichotoxin A with two additional alcohol groups and one additional branched methyl. Trichophycin A showed moderate cytotoxicity against neuro-2A cells and HCT-116 cells with EC_50_ values 6.5 ± 1.4 μM and 11.7 ± 0.6 μM, respectively. A hybrid tripeptide (peptide/polyketide) namely cryptomaldamide (**57**) was produced from *Moorea producens* [67]. Cryptomaldamide (**57**) consisted of four partial structures such as valine residue, unsaturated and methylated ketide-extended valine residue, serine group (hydroxy group), and CH_3_N_2_ (*N* methyl) group belonging to a guanidine carbon completing the fourth partial structure. The absolute configuration was established by Marfay’s method and determined to be l-configuration for *N*-methylvaline, valine and serine derivatives, while the vinyl methyl group with double bond was noted to be *E* configuration. Cryptomaldamide showed no activity against H-460 human lung cancer cells. Four unstaturated polyketide lactone derivatives, coibacins A–D (**58**–**61**) were isolated from *Oscillatoria* sp., Panama [68]. Spectroscopic analysis revealed the presence of *α*,*β*-unsaturated *δ*-lactone in all four compounds. The first two compounds (**58** and **59**) exhibited a methyl-substituted cyclo propyl ring, while the other two coibacin C and D (**60** and **61**) had methyl vinyl chloride moieties. Coibacin A (**58**) possessed two conjugated dienes separated by two methylene groups where the absolute configuration matched with *S* configuration of model *α*,*β-*unsaturated lactone ring while possessing a *trans* configured methyl cyclopropyl ring (S*, S*). Coibacin B (**59**) was found to be closely similar to coibacin A, excluding the two carbons which were absent in the dienes motif adjacent to the methyl cyclopropyl ring. Coibacin C (**60**) also contained similar feature as the above two compounds excluding the chlorine atom. Coibacin C possessed the same *α,β-*unsaturated *δ*-lactone with a difference observed in oxymethine, located adjacent to a methylene rather than an olefin, as well as two conjugated diene and a *bis*-allelic methylene connected with *β*-chloro and *α*-methyl substituents. Coibacin D (**61**) was similar to coibacin C (**60**), where coibacin D lack one of the two olefins forming the conjugated dienes in C (**60**). Moreover, the data analysis revealed the *E*-geometry of trisubstituted olefin in both C and D compounds. Coibacin A (**58**) showed potent activity against *Leishmania donovani* single amastigotes with an IC_50_ value of 2.4 μM. Coibacin D (**61**) exhibited potent cytotoxicity against NCI-H460 human lung cancer cells with an IC_50_ value of 11.4 μM, and coibacin B (**59**) exhibited anti-inflammatory activity in a cell based nitric oxide inhibition assay with an IC_50_ value of 5 μM (Figure 12, Table 2).

A polyhydroxy macrolide, bastimolide A (**62**) with a 40-membered ring was produced from *Okeania hirsuta*, collected from Panama [69]. Bastimolide A contained six partial structures including trisubstituted *α,β*-unsaturated lactone moiety, a *tert*-butyl group with (lactone methine, adjacent three methylenes, and distal secondary alcohol), 1,3,5-triol group and five 1,5-diol group. The absolute configurations of 1,5-diol and 1,3,5-triol group were assigned as *syn* and *anti/syn*, respectively. Bastimolide A exhibited potential antimalarial activity against four resistant pathogens of *Plasmodium falciparum* with IC_50_ values between 80 and 270 nM while exhibiting some toxicity to the control Vero cells with an IC_50_ value 2.1 μM. Polyhydroxylated 40 membered ring macrolactone, amentelides A (**63**) and B (**64**) were produced from a gray cyanobacterium near Puntan dos Amantes, Tumon Bay, Guam [70]. An *α,β*-unsaturated ester group, supported by a carbonyl, methyl and a vinyl group was noted. It was also confirmed that amentelides A and B comprised of a *tert*-butyl moiety, hence proving a one ring system with *Z* configuration. Amentelide B (**64**) was a monoacetylated analog containing an additional acetyl group instead of a hydroxyl group on position C-33 compared to amentelide A (**63**). Amentelide A exhibited potent antiproliferative activity in HT29 colorectal adenocarcinoma with an IC_50_ value of 0.87 ± 0.02 and HeLa cervical carcinoma cells with an IC_50_ value of 0.87 ± 0.07. Amentelide B (**64**) showed antiproliferative activity in HT29 colorectal adenocarcinoma with IC_50_ 12 ± 1.6 and HeLa cervical carcinoma cells with IC_50_ 9.9 ± 0.05. Another polyhydroxylated macrolactone, nuiapolide (**65**) was generated from *Okeania plumata*, near Lehua Rock [71]. The presence of an alkene or *α,β*-unsaturated ester group supported by carbonyl, methyl and a vinyl group as well as the presence of a *tert*-butyl group, and nine hydroxyl groups distributed throughout the saturated hydrocarbon ring structure was proved by spectroscopic analysis. Nuiapolide exhibited anti-chemotactive activity at concentrations as low as 1 μg/mL (1.3 μM) against Jurkat cells (human cancerous T lymphocyte cell line). A new cyclic polyketide-peptide hybrid namely Janadolide (**66**), was isolated from *Okeania* sp. collected near Janado, Okinawa [72]. Janadolide (**66**) consisted of two methyl groups, a *tert*-butyl group, vinyl methyl group, olefinic proton, six carbonyl groups and one double bond. Moreover, five amino acid units and one hydroxy unit like *N*-Me-Leu, *N*-Me-Ala, Gly, Val, Pro, and the presence of a residue derived from polyketide moiety (7-hydroxy-2,5,8,8-tetramethylnon-5-enoic) acid were noted. The olefinic bond in the polyketide moiety was established to be *E* configuration, and all amino acid residues were set to be l-configuration. Janadolide exhibited potent antitrypanasomal activity against *Trypanosoma bruceibrucei* with an IC_50_ value of 47 nM, which was stronger than the commonly used therapeutic drug, suramin (IC_50_ value of 1.2 μM). Jandolide showed no cytotoxicity against human cells.

**Figure 13:**
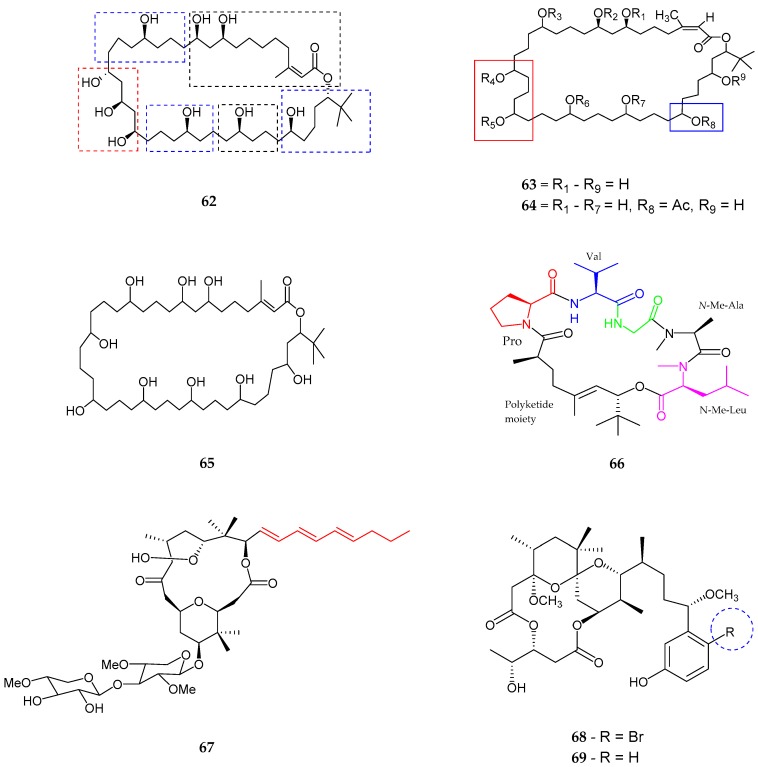
The chemical structures of: bastimolide A (**62**); amentelides A (**63**) and B (**64**); nuiapolide (**65**); janadolide (**66**); polycavernoside D (**67**); 3-methoxyaplysiatoxin (**68**); and 3-methoxydebromoaplysiatoxin (**69**).

A unique class of marine-derived 16 membered macrolide, polycavernoside D (**67**) was isolated from a red colored cyanobacterial *Okeania* sp. [73]. Polycavernoside D consisted of an allylic conjugated decanol triene, methylene with hydroxyl-methine, two terminal oxygenated methine, and two pentose pyranose rings with additional presence of two *gem*-dimethyl groups, ester linkage, the tetrahydropyran ring, tetrahydrofuran ring and *α*-hemiketal ketone group. Polycavernoside D exhibited moderate activity against H-460 human lung cancer cells with an EC_50_ value of 2.5 μM. In 2014, two new polyketides, 3-methoxyaplysiatoxin (**68**) and 3-methoxydebromoaplysiatoxin (**69**), analogs of aplysiatoxin, were produced from *Trichodesmium erythraeum*, from Singapore [74]. The absence of bromide ion found in 3-methoxydebromoaplysiatoxin (**69**) made it different from 3-methoxyaplysiatoxin (**68**). 3-methoxydebromoaplysiatoxin (**69**) showed significant activity against chikungunya virus in post-treatment of infected SJCRH30 cells with an EC_50_ value of 2.7 μM (Figure 13 and Table 2).

### 2.3. Alkaloids

Alkaloids are a chemically diverse group of naturally nitrogen-containing compounds that are produced by a large variety of marine microorganisms. Many alkaloids are pharmacologically well characterized and are mainly used as clinical remedies, ranging from chemotherapeutics to analgesic agents. Compared to all different types of natural compounds, alkaloids are identified by an enormous structural diversity with no uniform distribution. These marine alkaloids are frequently produced as potent toxins against predators. As such, more than thousands of alkaloids have been isolated from cyanobacteria.

A new hybrid thiazoline-containing alkaloid, laucysteinamide A (**70**), which was a monomeric analog of a disulfide-bonded dimeric compound, somocystinamide A was isolated from *Caldora penicillata,* collected at Lau Lau Bay Saipan, Northern Mariana Island [75]. The presence of an amide *N-*methyl group, a linear alkyl chain of eight sp^2^ carbons forming one mono-substituted vinylidene moiety, two di-substituted *trans* alkenes and an imine functional group with methyl thiazoline ring were revealed by spectroscopic techniques with the absolute configuration assigned as 2*R*. This compound was mildly cytotoxic to H-460 human non-small cell lung cancer cells with an IC_50_ value of 11 μM. Carriebowlinol [5-hydroxy-4-(chloromethyl)-5,6,7,8-tetrahydroquinoline] (**71**),was isolated from a non-polar extract of an abundant cyanobacterial mat collected on the coral reef at Carrie Bow Cay, Belize [76]. Analysis revealed the presence of one trisubstituted pyridine ring, a chloromethyl moiety (ClCH_2_), benzylic like secondary hydroxyl group, and the hydroxy methine connected to three methylene group (-CHOH-CH_2_-CH_2_-CH_2_-moeity) followed by the absolute configuration being (+)-5(*S*)-hydroxy-4-(chloromethyl)-5,6,7,8-tetrahydroquinoline. Carriebowlinol exhibited strong anti-fungal activity against *Dendryphiella salina, Lindra thalassiae*, and *Fusarium* sp. with IC_50_ values of 0.5 μM, 0.4 μM, and 0.2 μM, respectively. This compound also exhibited potent anti-bacterial activity against *Vibrio* sp.

**Figure 14:**
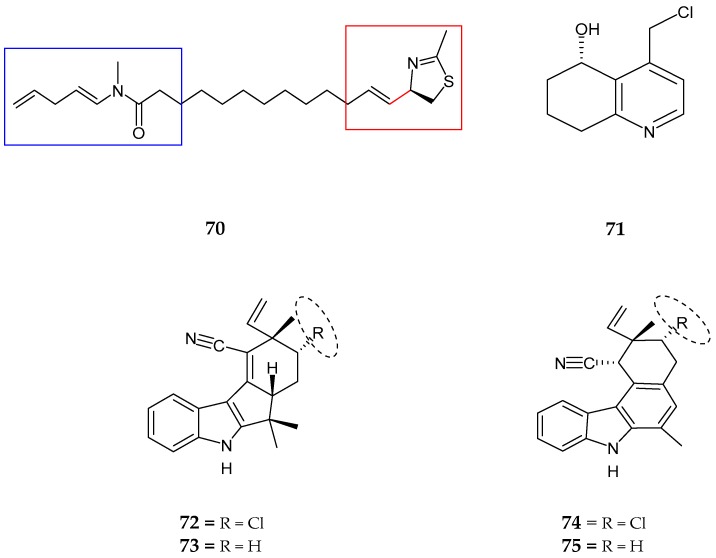
Chemical structures of: laucysteinamide A (**70**); carriebowlinol (**71**); 12-epi-fischerindole I (**72**) and deschloro 12-epi-fischerindole I nitrile (**73**); 12-epi-fischerindole W (**74**); and deschloro 12-epi-fischerindole W nitrile (**75**).

Four nitrile-containing fischerindole-type alkaloids, 12-epi-fischerindole I (**72**) and deschloro 12-epi-fischerindole I nitrile (**73**), 12-epi-fischerindole W (**74**) and deschloro 12-epi-fischerindole W nitrile (**75**) were obtained from the extract of *Fischerella* sp. [77]. 1,2 disubstituted indole moiety, a vinyl group with a terminal (*E* and *Z* isomers), three methyl groups, isonitrile unit, tetrasubstituted double bond and an isolated spin system consisting of two methines while connected by a methylene (CHCH_2_CH) from C-13 to C-15 were present in the structure (**72**). The relative absolute configuration was determined as 12R*, 13R*, and 15R*. The spectral data of deschloro 12-epi-fischerindole I nitrile (**73**) showed the absence of a chlorine atom, as well as a methine signal at C-15, was replaced by a methylene to form a spin system as CHCH_2_CH_2_ with the relative configuration being 12R* and 15R*. 12-epi-fischerindole W nitrile (**74**) possessed a six membered ring, which was not observed in all previously known fishcerindoles indicating that the sequence of the spin system was shortened comparatively to (**72**) and (**73**). The absolute configuration was established as 11R*, 12R*, and 13R*. Finally, deschloro 12-epi-fischerindole W nitrile (**75**) showed close similarity to (**68**) (3-methoxyaplysiatoxin) excluding the absence of chlorine atom. On the other hand, the difference observed at H-13 to H-14 (CH_2_CH_2_), indicated that the methine at H-13 in (**74**) was replaced by methylene in (**75**). The stereochemistry was noted to be 11R*, and 12R*. Deschloro 12-epi-fischerindole I nitrile (**73**) showed weak cytotoxic activity against HT-29 cells with an IC_50_ value of 23 µM (Figure 14, Table 3).

Three hapalindole-type alkaloids, namely hapalindole X (**76**), deschloro hapalindole I (**77**), and 13-hydroxy dechlorofontonamide (**78**), were isolated from the extract of two cultured cyanobacterium species; *Westiellopsis* sp. and *Fischerella muscicola* [78]. Spectral analysis of hapalindole X (**76**) confirmed the presence of an exomethylene moiety, a vinyl group and a terminal (*E*, *Z* isomers) including *gem*-dimethyl groups which together formed a seven-carbon spin system bearing configuration 10S*, 11R*, 13R*, and 15R*. Deschloro hapalindole I (**77**) was a derivative of the known hapalindole I and found to be closely similar to hapalindole X. The presence of a vinyl group with terminal *E* and *Z* isomers at the position of C-12 instead of exomethylene moiety proved the difference from other hapalindoles. The relative configuration was established to be 12R*, 15S*. Finally, the analysis revealed that 13-hydroxy dechlorofontonamide (**78**) was the hydroxyl derivative of previously known alkaloid structure dechlorofontonamide with the configuration being 12R*, 13S*, 15S*. Hapalindole X showed moderate cytotoxicity against HT-29 (colon), MCF-7 (breast), NCI-H460 (lung) and SF268 (CNS) cancer cells with IC_50_ values of 24.8, 35.4 ± 2.8, 23 ± 4.6, 23.5 ± 9.5µM, respectively. It also exhibited antimicrobial activity against *Mycobacterium tuberculosis* and *Candida albicans* with an IC_50_ value of 2.5 μM and moderate cytotoxicity in the Vero cell assay with an IC_50_ value of 35.2 μM.

**Figure 15:**
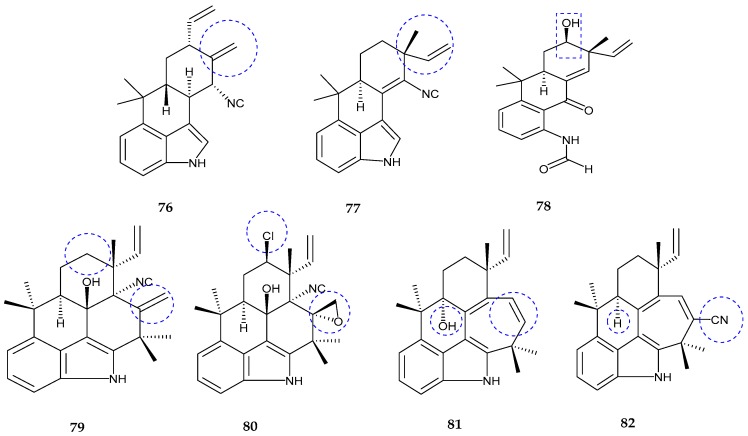
Chemical structures of: hapalindole X (**76**); deschloro hapalindole I (**77**); and 13-hydroxydechlorofontonamide (**78**); fischambiguines A (**79**) and B (**80**); ambiguine P (**81**); and ambiguine Q (**82**) nitrile.

Four hapalindole type alkaloids, namely fischambiguines A (**79**) and B (**80**), ambiguine P (**81**) and ambiguine Q nitrile (**82**), were produced from *Fischerella ambigua* [79]. Fischambiguines A (**79**) was confirmed to have 1,2,3-trisubstituted aromatic moiety, an exomethylene moiety, a vinyl group and a terminal (*E, Z* isomers) including five methyl groups and an isolated spin system CH_2_CH_2_CH from C-13 to C-15 along with the presence of an isonitrile unit at C-11 and a hydroxyl group at C-10. The configuration of fischambiguine A (**79**) was 10R*, 11R*, 12R*, and 15R*. Fischambiguine B (**80**) showed the presence of epoxy unit at C-25 and C-26 instead of an exomethylene group and a chlorine substitution at C-13 compared to A where the configuration was assigned as 10R*, 11S*, 12R*, 13R*, 15R* and 25R*. Ambiguine P (**81**) was an analog of ambiguine isonitriles where the noticed point was the absence of isonitrile unit with the absolute configuration being 12R* and 15R*. Ambiguine Q nitrile (**82**) showed the presence of an indole moiety, vinyl group and a terminal (*E, Z* isomers) including five methyl groups, tetrasubstituted double bond and an isolated spin system CH_2_CH_2_CH from C-13 to C-15 as well as isonitrile group. The configuration of ambiguine Q nitrile was seen as 12R* and 15R*. Fischambiguine A (**79**) exhibited antibiotic activities against *Mycobacterium tuberculosis*, *Staphylococcus aureus*, *Escherichia coli* and *Candida albicans* with MIC values of >100 µM, 82.3 µM, >100 µM and 15.3 µM, respectively. Fischambiguine B (**80**), exhibited activity against *Mycobacterium tuberculosis, Bacillus anthracis*, *Staphylococcus aureus*, *Mycobacterium smegmatis*, and *Escherichia coli* with MIC values of 2.0, 28.7, 19.4, 23.4, and >100 µM, respectively. It also showed inhibitory cytotoxicity against Vero cells with an IC_50_ value of >128 µM. Ambiguine P nitrile (**81**) revealed antimicrobial activity against *Mycobacterium tuberculosis*, *Staphylococcus aureus*, and *Candida albicans* with MIC values of >100, >100 and 32.9 µM, respectively (Figure 15, Table 3).

### 2.4. Lipids

Fatty acid amides are one of the typical lipophilic class of compounds, which are highlighted by the presence of an amide bond in a fatty acid chain and in some cases with incorporated halogen atoms. Since the last two decades, an assortment of fatty acid amides have been produced from marine cyanobacteria including pitiamide A with their analogs 1*E*-pitiamide B and 1*Z*-pitiamide B, serinolamide A, propenediester, bartolosides, columbamides A–C, kimbeamides, kalkitoxin, taveuniamides, besarhanamides and credneramides.

**Figure 16:**
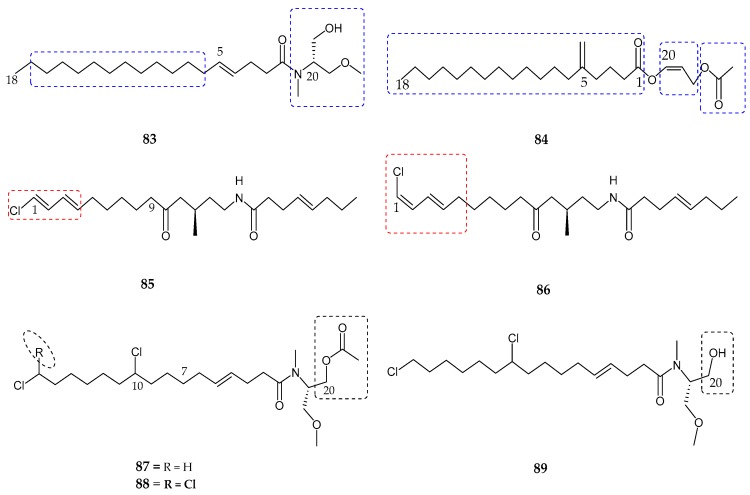
Chemical structures of: serinolamide A (**83**); propenediester (**84**); 1*E*-pitiamide B (**85**); 1*Z*-pitiamide B (**86**); and columbamides A–C (**87**–**89**).

In 2011, two new endocannabinoid-like lipids, namely serinolamide A (**83**) and propenediester (**84**), were identified [80] and isolated from the extracts of *Lyngbya majuscula* and *Oscillatoria* sp. collected at Papua New Guinea and Panama, respectively. The structure of serinolamide A (**83**) showed the presence of three methyl groups, a monounsaturated alkyl chain, hydroxyl and amide-carbonyl groups as well as a 1st partial structure being saturated fatty acid moiety and a 2nd substructure consisting of a dimethyl serinol moiety, where the linkage in between oxygen-bonded methylenes with nitrogen group was observed. Analysis of propenediester (**84**) revealed the presence of two methyl groups at both ends, ethyl groups, two ester groups, and a saturated fatty acid chain with 16 methylenes. Both compounds (**83**) and (**84**) were evaluated for human cannabinoid receptors (G protein coupled-receptor) in radioligand binding assays. Serinolamide A (**83**) exhibited moderate affinity for the CB_1_ receptor with an IC_50_ value of 2.3 ± 0.1 µM, and found inactive for the CB_2_ receptor with an IC_50_ value of >10 µM. Two geometric isomers/analogs related to previously known pitiamide A, namely 1*E*-pitiamide B (**85**) and 1*Z*-pitiamide B (**86**), were isolated from marine cyanobacterium sp. in the Mariana Islands [81]. 1*E*-pitiamide B (**85**) contained a ketone group, an ester linkage, two methyl groups, a terminally chlorinated conjugated diene, and an isolated C–C double bond with three partial structures. However, the terminally chlorinated conjugated diene of compound (**86**) observed as *Z-E* configuration differentiated it from that of (**85**). Both compounds (**85**) and (**86**) were evaluated for their anti-proliferative effects against HCT116 colorectal cancer cells with IC_50_ values of 5.1 µM and 4.5 µM, respectively. A new class of di- and tri-chlorinated acyl amides cannabinomimetic compounds namely columbamides A–C (**87**–**89**) were produced from the cultured extract of cyanobacterium species [82]. Columbamide A (**87**) revealed the presence of *N*-methyl amide adjacent to a carbonyl group, *O*-methyl group, an acetoxy group, and an alkyl chain as well as two chlorine atoms. Columbamide B (**88**) was an analog of columbamide A where the only difference observed between both was the presence of an additional chlorine atom at the terminal C-16, which in turn showed a terminal *gem*-dichloro functionality. On the other hand, columbamide C (**89**) lack an acetoxy group at the C-20 position compared to columbamide A (**87**). The absolute configuration of the dimethylated and acetylated serinol residue was established by Marfay’s method, as d- and l-*N*-methyl-*O*-methylserinol. Both compounds A and B exhibited potent affinity for the CB_1_ receptor as well as for the CB_2_ receptor with IC_50_ values of 0.59 ± 0.08 µM, 0.41 ± 0.06 µM and 1.03 ± 0.12 µM, 0.86 ± 0.09 µM, respectively (Figure 16, Table 4). 

**Figure 17:**
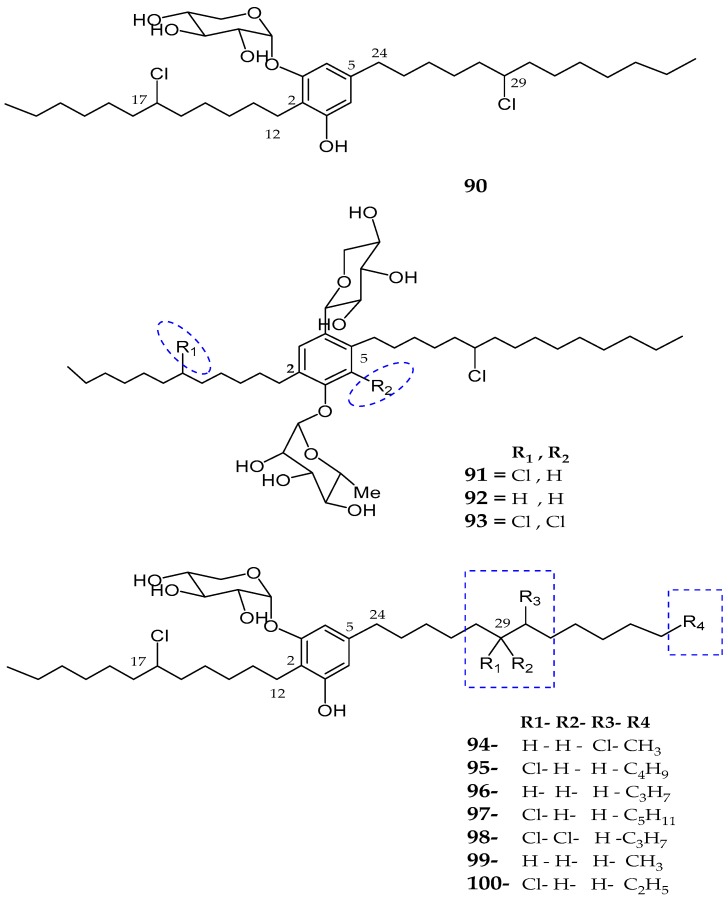
Chemical structures of: bartolosides A–D (**90**–**93**); and bartolosides E–K (**94**–**100**).

Four new glycosylated and halogenated dialkylresorcinol glycolipids, namely bartolosides A–D (**90**–**93**), were isolated from the crude extract of *Nodosilinea* sp. and *Synechocystis salina* sp. [83]. The presence of an aromatic/resorcinol ring, phenol group, glycosidic linkage (*O*-linkage), *β*-xylopyranosyl moiety, two methyl groups, and two chlorinated alkyl chains with a propyl substituted at one end of the linear chain (CH_2_CH_2_CH_3_) was seen for bartoloside A (**90**). Bartoloside B (**91**) showed close similarity to bartoloside A (**90**) with a difference in the two sugar moieties including xylose and rhamnose. The absolute configuration of the sugar units was established as d-xylose and l-rhamnose. The difference observed in bartoloside C (**92**) was an absence of a substituted chlorine atom at CH-17 in the alkyl chain, while bartoloside D (**93**) bear an additionally substituted chlorine atom in the aromatic ring as compared to bartoloside B (**91**). Seven new glycolipids analog of bartoloside A, namely bartolosides E–K (**94**–**100**) were isolated from *Synechocystis salina* strain LEGE 060099, collected in Northern Portugal [84]. Spectroscopic data of bartolosides E–K (**94**–**100**) revealed that most of the regions were found highly similar to bartoloside A with different alkyl chain lengths or halogenation patterns. Bartoloside A (**90**) and E–K (**94**–**100**) showed cytotoxic activities against human cancer cell lines including bone osteosarcoma (MG-63), colon carcinoma (RKO), and mammary gland ductal carcinoma (T-47D) with an IC_50_ value of >10 µM. Moreover, bartoloside A and E were active against MG-63 cells, RKO cells, and T-47D with IC_50_ values of 22 and 39 µM, 40 and 40 µM, and 23 and 22 µM, respectively (Figure 17, Table 4).

## 3. Applications

Cyanobacteria have been recognized as a productive source of secondary metabolites with possible biotechnological applications in the pharmacological area. Recently, production of natural products with commercial and medical applications has also heightened interest in investigating these organisms [20,85]. In fact, cyanobacteria have the ability to simultaneously produce potent toxins as well as infinite metabolites that have been praised for their antibacterial, antifungal, immunosuppressive, anticancer and anti-tuberculosis activities [86]. Associated secondary metabolites are from all classes including peptides, polyketides, alkaloids, terpenoids, and lipids. For example, tesiamide A and B exhibited cytotoxic activity against human nasopharyngeal carcinoma cell lines because of the presence of unique amino acid residue, 4-amino-3-hydroxy-5-phenylpentanoic acid and their chemically related analogs exhibited inhibitory activities against human nasopharyngeal carcinoma and non-small cell lung tumor cell lines [87,88,89]. Gamma linolenic acid (GLA), an important fatty acid in the human body, is converted into arachidonic acid and then into prostaglandin E2, involved in lowering blood pressure which is also important in lipid metabolism [90]. Prominently, brentuximab vedotin (trade name Adcetris), marine peptide-derived medicine, was approved by the U.S. Food Drug Administration (FDA) for the treatment of Hodgkin lymphoma and anaplastic large cell lymphoma [31]. It was also observed that cryptophycins, from *Nostoc* sp., were thousand times more potent than the current anticancer medications. Moreover, dolastatin 10 and its chemically associated analog, symplostatin, were potent microtubule polymerization inhibitors that had the ability to inhibit cancer growth [90,91]. Likewise, dolastatin 15, another member of dolastatin family, served as a weak tubulin inhibitor by adhering to the vinca region of tubulin and induced apoptosis through Bcl-2 phosphorylation in several malignant cell types. However, no clinical trial has been initiated with this compound because of its structural complexity, little synthetic yield, and poor water solubility [91].

An individual family of indole monoterpene alkaloids, namely welwitindolinones B and C, originally produced by true-branching heterocystous filamentous cyanobacterium *Hapalosiphon welwitschii*, were noted as potent antitumor agents that exhibited anti-mitotic activity with multidrug resistance (MDR) reversal properties [92]. Pitiamide A and its two analogs showed an anti-proliferative effect on HCT116 cells. Pitiamide A was tested individually and caused membrane hyperpolarization and the increase of intracellular calcium in HCT116. Specific cannabinoid receptors (CB_1_ and CB_2_) in central and peripheral mammalian tissues had been discovered due to an important development in neuropharmacological research [93,94]. Only few cannabinoids related medicinal products are available on the market and, as such, Sativex was introduced as the first medicine in this type of class [95]. Moreover, the National Cancer Institute had declared that a fat solvable photosynthetic pigment, β-carotene was anti-carcinogenic in nature. Eventually, it is beneficial to decrease the risk of heart infections by regulating the cholesterol level.

## 4. Conclusions

This review presented a survey of the wide variety of recently published secondary metabolites from marine cyanobacteria highlighting peptides, polyketide, alkaloid, lipid parts and any combination thereof. Approximately 100 marine cyanobacterial metabolites were explained here with some focus on multiple attractive structural characteristics including amino acid residues, terminal fatty acyl residues (Dhoya, Hmoya, Amoya, etc.) and heterocyclic aromatic moieties such as thiazoline, chlorinated or dechlorinated alkyl linear chains and resorcinol rings. These underlined different features created not only structural diversity but also contributed significantly to the biological activities. Nevertheless, some of these metabolites were not of interest due to their weak bioactivities, but most of them displayed significant biological properties such as anticancer, antitumor, antimalarial, antifungal, antibacterial, and anti-inflammatory. The recently reported compounds with potent activities were: wewakzole B, kohamamide, samoamide, odobromoamide, uramamide, caldoramide, janadolide, laucysteinamide, trichophycin A and dudawalamides.

Moreover, carriebowlinol, balticidins, and glycosylated hassallidins were found to be active against pathogenic fungi, which fascinated researchers in the pharmaceutical area due to a rise in the percentage of invading fungal infections and resistance to several currently accepted drugs. It was also observed that cyanobacteria could be easily cultured but complications arose during the isolation and elucidation processes of compounds due to the presence of partial structures to establish complex structures. As a whole, with the combination of knowledge from various fields, natural products from marine cyanobacteria should be regarded as a prolific source for biotechnological development and applications for pharmaceutical purposes.

## Figures and Tables

**Table 1:** Bioactive secondary metabolites isolated from cyanobacteria in peptide class.

Compounds	Species	Bioactivity	References
Urumamide (**1**)	*Okeania* sp.	Anticancer	[32]
Medusamide A (**2**)	Cyanobacterial sp.	No cytotoxicity	[33]
Odoamide (**3**)	*Okeania* sp.	Brine shrimp toxicity	[34]
Bouillonamide (**4**)	*Moorea bouillonii*	Antitumor	[35]
Companeramides A–B (**5**–**6**)	Cyanobacterial assemblage	Antiplasmodium	[36]
Dudawalamides A–D (**7**–**10**)	*Moorea producens*	Antiplasmodium and Antiparasitic	[37]
Kohamamides A–C (**11**–**13**)	*Okeania* sp.	Weak against Cancer	[38]
Kohamamide B (**12**)	*Okeania* sp.	Potent cytotoxicity	[38]
Viequeamide A–B (**14–15**)	*Rivularia* sp.	Potent anticancer activity	[39]
Pitiprolamide (**16**)	*Lyngbya majuscula*	Weak cytotoxic activity	[40]
Pitipeptolide C–E (**17**–**19**)	*Lyngbya majuscula*	Weak cytotoxic activity	[41]
Pitipeptolide (F) (**20**)	*Lyngbya majuscula*	Potent microbial activity	[41]
Apratoxin H (**21**)	*Moorea producens*	Anticancer activity	[42]
Apratoxin A sulfoxide (**22**)	*Moorea producens*	No cytoxicity	[42]
Samoamide A (**23**)	*Symploca* sp.	Anticancer activity	[43]
Wewakzole B (**24**)	*Moorea producens*	Anticancer activity	[44]
Odobromoamide (**25**)	*Okeania* sp.	Antitumor activity	[46]
Anabaenopeptins NP 883, 869, 867, 865, 813 (**26**–**30**)	Cyanobacterial sp.	No cytotoxicity	[47]
Maedamide (**31**)	*Lyngbya* sp.	Strong anti-chymotrypsin	[48]
Caldoramide (**32**)	*Caldora penicillata*	Antitumor	[49]
Tasiamides C–E (**33**–**35**)	*Symploca* sp.	No cytotoxicity	[50]
Tesiamide F (**36**)	*Lyngbya* sp.	Anti-proteolytic activity	[51]
Balticidin A–D (**37**–**40**)	*Anabaena cylindrica*	Antifungal	[52]
Hassallidins C–D (**41**–**42**)	*Anabaena* sp.	Anti- fungal	[53]
Kurahyne (**43**)	*Lyngbya* sp.	Anti-cancer	[55]
Kurahyne B (**44**)	*Lyngbya* sp.	Anti-cancer	[56]
Jahanyne (**45**)	*Lyngbya* sp.	Anti-tumor activity	[57]
Kanamienamide (**46**)	*Moorea bouillonii*	Antitumor	[58]
Biseokeaniamides A–C (**47**–**49**)	*Okeania* sp.	inhibited sterol *O*-acyltransferase SOAT1 and SOAT2	[59]
Maylngamide (**50**)	*Moorea producens*	Cytotoxic	[60]
Malyngamide Y, (+)-floridamide (**51**–**52**)	*Moorea producens*	Anticancer	[61]
Malyngamide 4 (**53**)	*Moorea producens*	Anticancer	[62]
Almiramide D (**54**)	*Oscillatoria nigroviridis*	Potent brine shirimp toxicity	[63]
Hoshinolactam (**55**)	Cyanobacterial sp.	Anti-trypanosomal activity	[64]

**Table 2:** Bioactive secondary metabolites from cyanobacteria in polyketide class.

Compound	Species	Bioactivities	References
Trichophycin A (**56**)	*Trichodesmium thiebautii*	Anti-cancer	[65]
Cryptomaldamide (**57**)	*Moorea producens*	No Cytotoxicity	[67]
Coibacins A–D (**58**–**61**)	*Oscillatoria* sp.	Anti-parasite, cancer and inflammatory	[68]
Bastimolide A (**62**)	*Okeania hirsuta*	Antimalarial activity	[69]
Amentelides A–B (**63**–**64**)	Gray cyanobacterium	Anti-Cancer	[70]
Nuiapolide (**65**)	*Okeania plumata*	Anti-cancer	[71]
Janadolide (**66**)	*Okeania* sp.	Anti-trypanasomal against *Trypanosoma brucei brucei*	[72]
Polycavernoside D (**67**)	*Okeania* sp.	Anti-cancer	[73]
3-methoxyaplysiatoxin and 3-methoxydebromoaplysiatoxin (**68**–**69**)	*Trichodesmium erythraeum*	Anti-viral	[74]

**Table 3:** Bioactive secondary metabolites from cyanobacteria in alkaloid class.

Compounds	Species	Bioactivities	References
Laucysteinamide A (**70**)	*Caldora Penicillata*	Anticancer	[75]
Carriebowlinol (**71**)	Cyanobacterial mat	Anti-fungal	[76]
12-epi-fischerindole I (**72**)	*Fischerella* sp.	No cytotoxicity	[77]
Deschloro 12-epi-fischerindole I (**73**)	*Fischerella* sp.	Anti-cancer	[77]
12-epi-fischerindole W (**74**)	*Fischerella* sp.	No cytotoxicity	[77]
Deschloro 12-epi-fischerindole W (**75**)	*Fischerella* sp.	No cytotoxicity	[77]
hapalindole X (**76**)	*Westiellopsis* sp.	Anti-cancer and anti-microbial	[78]
deschloro hapalindole I (**77**)	*Fischerella muscicola*	Anti-cancer and anti-microbial	[78]
13-hydroxydechlorofontonamide (**78**)	*Westiellopsis* sp. and *Fischerella muscicola*	Anti-cancer and anti-microbial	[78]
fischambiguines A–B (**79**–**80**), Ambiguine P (**81**) and Ambiguine Q (**82**)	*Fischerella ambigua.*	Anti-tuberculosis	[79]

**Table 4:** Bioactive secondary metabolites from cyanobacteria in lipid class.

Compound	Species	Bioactivity	References
Serinolamide A (**83**)	*Lyngbia majuscula*	Moderate affinity for CB_1_ receptor	[80]
Propenediester (**84**)	*Oscillatoria* sp.	No affinity	[80]
1*E*-pitiamide B (**85**)	Cyanobacterial sp.	Anti-cancer	[81]
1*Z*-pitiamide B (**86**)	Cyanobacterial sp.	Anti-cancer	[81]
Columbamides A–C (**87**–**89**)	Cyanobacterial sp.	Potent affinity for CB_1_ and CB_2_ receptors	[82]
Bartolosides A–D (**90**–**93**)	*Nodosilinea* sp. and *Synechocystis salina* sp.	Not reported	[83]
Bartolosides E–K (**94**–**100**)	*Synechocystis Salina*	No cytotoxicity	[84]
